# Expression and Evolution of the Non-Canonically Translated Yeast Mitochondrial Acetyl-CoA Carboxylase Hfa1p

**DOI:** 10.1371/journal.pone.0114738

**Published:** 2014-12-11

**Authors:** Fumi Suomi, Katja E. Menger, Geoffray Monteuuis, Uta Naumann, V. A. Samuli Kursu, Antonina Shvetsova, Alexander J. Kastaniotis

**Affiliations:** Faculty of Biochemistry and Molecular Medicine and Biocenter Oulu, University of Oulu, Oulu, Finland; Simon Fraser University, Canada

## Abstract

The *Saccharomyces cerevisiae* genome encodes two sequence related acetyl-CoA carboxylases, the cytosolic Acc1p and the mitochondrial Hfa1p, required for respiratory function. Several aspects of expression of the *HFA1* gene and its evolutionary origin have remained unclear. Here, we determined the *HFA1* transcription initiation sites by 5′ RACE analysis. Using a novel “Stop codon scanning” approach, we mapped the location of the *HFA1* translation initiation site to an upstream AUU codon at position −372 relative to the annotated start codon. This upstream initiation leads to production of a mitochondrial targeting sequence preceding the ACC domains of the protein. *In silico* analyses of fungal *ACC* genes revealed conserved “cryptic” upstream mitochondrial targeting sequences in yeast species that have not undergone a whole genome duplication. Our Δ*hfa1* baker's yeast mutant phenotype rescue studies using the protoploid *Kluyveromyces lactis ACC* confirmed functionality of the cryptic upstream mitochondrial targeting signal. These results lend strong experimental support to the hypothesis that the mitochondrial and cytosolic acetyl-CoA carboxylases in *S. cerevisiae* have evolved from a single gene encoding both the mitochondrial and cytosolic isoforms. Leaning on a cursory survey of a group of genes of our interest, we propose that cryptic 5′ upstream mitochondrial targeting sequences may be more abundant in eukaryotes than anticipated thus far.

## Introduction

A fairly recently recognized feature of mitochondria, conserved in all eukaryotes, is their ability to synthesize fatty acids in an acyl carrier protein dependent manner. The mitochondrial fatty acid synthesis (mtFAS) pathway is best described in the yeast *Saccharomyces cerevisiae*, where the disruption of the pathway results in a respiratory deficient phenotype, lack of cytochromes, and loss of or decrease in lipoic acid. There is overwhelming evidence that lipoic acid is one of the downstream products of mtFAS, an enzyme cofactor essential for the function of several mitochondrial enzyme complexes [1], [2]. The mtFAS process has been proposed to act as a regulatory circuit, controlling mitochondrial biogenesis in response to acetyl-CoA availability, and the presented evidence suggests a physiological role for fatty acids products of mtFAS longer than the octanoic acid precursor of lipoic acid in this regulatory circuit [3]. Acetyl-CoA carboxylase (ACC) catalyses the ATP-dependent carboxylation of acetyl-CoA to form malonyl-CoA, the first committed step in FAS. This reaction product is an intermediate in the *de novo* synthesis of long-chain fatty acids both in mitochondria and cytosol. It is also a substrate for distinct fatty acyl-CoA elongation enzymes in the endoplasmic reticulum. In mammals and probably also in other metazoans, malonyl-CoA is a transmitter of a signaling cascade sensing nutrition status of the body and tissue and controls the transport of long-chain fatty acids into mitochondria for β-oxidation by inhibiting carnitine palmitoyltransferaseI (CPT1) [4]. In key tissues, the regulation of the rate of β-oxidation plays a major role in orchestrating whole body metabolic adaptations to changes in nutrient availability and to fuel traffic between organelles. There are still several intriguing questions surrounding the origin and expression of mitochondrial matrix ACC in yeast (*HFA1*) as well as higher eukaryotes.

In *S. cerevisiae*, Acc1p is found to be the cytosolic version of acetyl-CoA carboxylase [5] while Hfa1p represents the mitochondrial counterpart [6]. Translation initiated from the first ATG start codon of the *HFA1* ORF does not produce a functional enzyme, and homology to *ACC1* extends far towards the 5′ direction of the annotated ATG of *HFA1* [6]. Further analysis for this upstream region revealed a putative mitochondrial import sequence required for mitochondrial function. The only additional, far upstream in-frame ATGs are almost immediately followed by stop codons, requiring exotic mechanisms like mRNA editing or ribosomal frame-shifting to allow translation of *HFA1.* It has been recognized already in the 1980s that yeast is capable to initiate gene product translation from non AUG start codons [7], and a physiological role of translation from non-canonical initiation sites in the production of mitochondrially localized protein isoforms of several tRNA synthetases in *S. cerevisiae* [8]–[11] has been well documented. A perhaps more feasible solution to the problem of *HFA1* expression would therefore be a non AUG start codon in *HFA1* translation as has been previously proposed [6]. To date, there are large discrepancies in the existing reports for the transcription and translation start sites of *HFA1*.

In addition to the question of *HFA1* expression, the evolutionary origin of this gene and its *ACC1* counterpart has remained unresolved. Kellis *et al.* used the *ACC1*/*HFA1* gene pair as an example for gene specialization after the genome duplication that led to the speciation of *S. cerevisiae.* The authors argued for the gain of a mitochondrial import sequence by the gene that was to become *HFA1* after duplication of the proto-fungal ACC [12]. The discussion on the evolution of *HFA1* by Hoja *et al*. can also be interpreted in a similar manner. In contrast, Turunen *et al*. suggested the common ancestor of both genes to be a one-gene variant encoding both the cytosolic and mitochondrial proteins, the former initiated from a canonical ATG translation start codon and the latter from a non-AUG triplet [13], but *in vivo* experimental support for this hypothesis is lacking.

In this study, we identified the transcription and translation initiation sites to provide insight into how the expression of *HFA1* is achieved. We have used *Kluyveromyces lactis*, a protoploid yeast strain closely related to the degenerate diploid *S. cerevisiae* and commonly used for comparative genetic studies, to address the question of the evolutionary origin of yeast acetyl-CoA carboxylases. The presence of cryptic 5′ untranslated regions (UTRs) containing mitochondrial targeting sequences in yeast raises the intriguing question about the possibility of the presence of a hidden cache of low abundance eukaryotic mitochondrial proteins that have not been recognized due to a primary function in another compartment.

## Materials and Methods

### Yeast Strains, media and genetic methods

Wild type and mutant strains of *S. cerevisiae* and sources are summarized in Table 1. Media used are as follows: YPD [1% yeast extract (DIFCO, NJ, USA), 2% Bacto- peptone (DIFCO), 2% glucose], SCD/-Uracil [0.67% Yeast Nitrogen Base without amino acids (DIFCO), 0.19% Synthetic complete drop out mix without uracil (SIGMA-Aldrich, St. Louis, MO, USA), 2% glucose], SCD[0.67% Yeast Nitrogen Base without amino acids, 0.19% Synthetic complete drop out mix without uracil, 0.008% Uracil, 2% glucose], SCG[0.67% Yeast Nitrogen Base without amino acids, 0.19% Synthetic complete drop out mix without uracil, 0.008% Uracil, 3% glycerol], SCL[0.67% Yeast Nitrogen Base without amino acids, 0.19% Synthetic complete drop out mix without uracil, 0.008% Uracil, 1% D/L lactic acid]. SCD -Leucine [0.67% Yeast Nitrogen Base without amino acids, 0.16% Synthetic complete drop out mix without leucine, 2% glucose]. Solid media was prepared with 2%agar.

**Table 1:** Yeast strains used for this study.

Strain	Genotype	Reference
W1536 5B	*MAT* ***a*** *;* Δ*ade2;* Δ*ade3; his3-11; his3-12; leu2-3; trp1-1; ura3-1*	H. Zou and R. Rothstein [27] [43]
W1536 8B	*MATα;* Δ*ade2;* Δ*ade3; his3-11; his3-12; leu2-3; trp1-1; ura3-1*	H. Zou and R. Rothstein [27] [43]
BY4741 Δ*hfa1*	*MAT* ***a*** *;* Δ*his3-1;* Δ*leu2-0;* Δ *met15-0;* Δ*ura3-0; YMR207c::kanM4*	Euroscarf
BY4742 Δ*hfa1*	*MATα;* Δ *his3-1;* Δ*leu2-0;* Δ*lys2-0;* Δ*ura3-0; YMR207c::KanMX*	Euroscarf
W1536 8B Δ*hfa1* pTSV30 *HFA1*	*MATα;* Δ*ade2;* Δ*ade3; his3-11; his3-12; leu2-3; trp1-1; ura3-1; YMR207c::kanMX4* pTSV30 *HFA1*	This study
W1536 8B Δ*hfa1*	*MATα;* Δ*ade2;* Δ*ade3; his3-11; his3-12; leu2-3; trp1-1; ura3-1*	This study
W1536 8B Δ*etr1*	*MATα;* Δ*ade2;* Δ*ade3; his3-11; his3-12; leu2-3; trp1-1; ura3-1; mrf::kanMX*	This study

### Construction of plasmids

Plasmids used for this study are listed in Table 2. Because *HFA1* is a very large gene, a PstI site was introduced in position +2449 (relative to the annotated ATG) to allow easier handling. The PstI site does not change the encoded amino acid sequence. To generate this construct, the sequence encoding the C-terminal end of *HFA1* was PCR amplified from wild type yeast genomic DNA using oligonucleotides *HFA1* +2449 5′PstI and *HFA1* clon/KO 3′SmaI and cloned into the PstI and SmaI sites of YCp*lac33* to generate YCp33*HFA1* C-term. The N-terminal end of *HFA1* was PCR amplified using oligonucleotids *HFA1* -980 5′HindIII and *HFA1* +2449 3′PstI and ligated into YCp33*HFA1* C-term to generate YCp33*HFA1* with *HFA1* gene under its own regulatory sequences. The YCp33*hfa1-1* was generated like YCp33*HFA1*, but the sequence encoding the N-terminal part of *hfa1-1* gene was PCR amplified from a synthetic petite mutant [3] and contained the C273T mutation, leading to the premature stop codon Q91STOP. The bluescript vector pBS KS (+) was digested with *HindIII* and *PstI* (New England Biolabs 20U/µl). The N-terminal part of *HFA1* was cut out from YCp33 *HFA1* with *HindIII* and *PstI* (New England Biolabs 20U/µl), inserted to the digested bluescript vector pBS KS (+). This plasmid was used for the site directed mutagenesis manipulations.YCp33 *HFA1 -273,* YCp33 *HFA1 -282,* YCp33 *HFA1-312,* YCp33 *HFA1 -360,* YCp33 *HFA1 -363*, YCp33 *HFA1 -372*, YCp33 *HFA1 -375*, YCp33 *HFA1 -381* and YCp33 *HFA1 -378* were generated by site-directed mutagenesis (QuikChange XL Site-Directed Mutagenesis Kit, Stratagene, La Jolla, California, USA) of pBS KS N-*HFA1* (Table 2). *HFA1* with point mutation was cloned into the expression vector YCp33 *HFA1* (Table 2) using the PstI and HindIII restriction sites.

**Table 2:** Plasmids used for the acetyl-CoA carboxylases study in yeasts.

Plasmid	Description	Reference
YCp*lac*33	Single copy vector, empty	[15]
YCp33 *HFA1*	Full length *HFA1*with and engineered Pst1 site in position +2449, cloned into *HindIII* and *SmaI* restriction sites	This study
pET23a+		Novagen (Merck Chemicals, Nottingham, United Kingdom)
pET23a+ *HFA1* tx start	Promoter region of *HFA1* cloned within *BamH1* and *XbaI* restriction sites (see description in text)	This study
YEp*lac*181	Single copy vector, empty	[15]
pBS KS +		Fermentas (Helsinki, Finland)
pBS KS N-*HFA1*		This study
pTSV30A	Multi copy vector, empty	[27]
pTSV30*HFA1*	Full length *HFA1* cloned into *XmaI* and *XbaI* restriction sites	This study [44]
YCp33 *HFA1* −381	Generated through site directed mutagenesis from YCp33 *HFA1* or pBS N-*HFA1* using the appropriate primers	This study
YCp33 *HFA1* −378	Generated through site directed mutagenesis from YCp33 *HFA1* or pBS N-*HFA1* using the appropriate primers	This study
YCp33 *HFA1* −375	Generated through site directed mutagenesis from YCp33 *HFA1* or pBS N-*HFA1* using the appropriate primers	This study
YCp33 *HFA1* −372	Generated through site directed mutagenesis from YCp33 *HFA1* or pBS N-*HFA1* using the appropriate primers	This study
YCp33 *HFA1* −363	Generated through site directed mutagenesis from YCp33 *HFA1* or pBS N-*HFA1* using the appropriate primers	This study
YCp33 *HFA1* −360	Generated through site directed mutagenesis from YCp33 *HFA1* or pBS N-*HFA1* using the appropriate primers	This study
YCp33 *HFA1* −312	Generated through site directed mutagenesis from YCp33 *HFA1* or pBS N-*HFA1* using the appropriate primers	This study
YCp33 *HFA1* −282	Generated through site directed mutagenesis from YCp33 *HFA1* or pBS N-*HFA1* using the appropriate primers	This study
YCp33 *HFA1* −273	Full length *HFA1* cloned into *HindIII* and *SmaI* restriction sites PCR amplified from genomic DNA of *HFA1* point mutant (Kursu *et al.* 2013)	This study
Ycp33 *ADH1* promoter	ADH promoter cloned into *HindIII* and *XbaI* restriction sites	This study
YCp33 *K.lactis HFA1* *	Full length *K.lactis HFA1* with upstream sequence predicted to be MTS cloned into *SpeI* and *EcoRI* restriction sites	This study
YCp33 *K.lactis HFA1* * *noMTS*	Full length *K.lactis HFA1* cloned into *SpeI* and *EcoRI* restriction sites	This study

*these plasmids are unstable in *E. coli* and have to be maintained in *S. cerevisiae* frozen stocks

Plasmid YCp33p*ADH1* was constructed by amplification of the −720 to −12 region of the *ADH1* promoter sequence (numbers are location upstream relative to the initiation ATG of the *ADH1* ORF) from yeast genomic DNA using primers HindIII*ADH1*prom 5′ and *ADH1* prom XbaI 3′ and ligation of this product digested with HindIII and XbaI into the corresponding sites of YCp*lac*33.

The bluescript vector pBS KS (+) was digested with *HindIII* and *PstI* (New England Biolabs 20U/µl). The N-terminal part of *HFA1* was cut out from YCp33 *HFA1* with *HindIII* and *PstI* (New England Biolabs 20U/µl), inserted to the digested bluescript vector pBS KS (+).

### Oligonucleotides

The DNA oligonucleotides designed for this study and their purpose are listed in Table 3.

**Table 3:** Oligonucleotides used for the acetyl-CoA carboxylases study in yeasts.

Name	Sequence
*HFA1* +2449 5′PstI	AAACTGCAGCCACTTCTTAAAATTAGTGAA
*HFA1* clon/KO 3′SmaI	CTCTCAACCAATGTCACCAA
*HFA1* −980 5′HindIII	CCCAAGCTTGTTGTACACAGCAGAGTG
*HFA1* +2449 3′PstI	AAACTGCAGGGCAACATAAACTACATGAT
*BamH1* Hfa1p transcr. Start 5′	TTATGGATCCGCATTCTGAAAGTGAGATAG
*Xba1* Hfa1p transcr. Start 3′	TTATTCTAGAAGAGAGGTTCCTACTACTTC
Hfa1p transcr. Start 3′ seq	AGAGGTTCCTACTACTTC
Hfa1p 5′RACE Sp1	ATCATTGAACGTCTCGTACGC
Hfa1p 5′ RACE Sp2	CTTTAGATCTCTTTCTTTCACCGCAGCA
Hfa1p 5′ RACE Sp3	CGAGATGAATGCCTATACG
*Xba1* Hfa1p transcr. Start 3′ new	TTATTCTAGACGCGAGCTTGTACTGTCCTATTCG
HA-His tag coding *Sph1*	GTACCCATACGACGTCCCAGACTACGCTCATCATCATCATCATCATT AGGCATG
HA-His tag complementing *Pst1*	CTAATGATGATGATGATGATGAGCGTAGTCTGGGACGTCGTATGGGTACTGCA
*Hfa1*-Ile127 *Pst1*	TTATCTGCAGGATACACGGGGATTTTAGCGC
*Hfa1*-Arg135 *Pst1*	TTATCTGGCAGTCTATATGTGAACCAGACCAAG
*Hfa1*-prom-5′ *EcoR1*	TTATGATATCCGTTCTTCGATACCCTGTCGAA
*HFA1* Stop −381 5′-3′	CCGGATACATTCTCACTTTTACATTACCTAATTCACAATTACTTG
*HFA1* Stop −381 5′-3′	CAAGTAATTGTGAATTAGGTAATGTAAAAGTGAGAATGTATCCGG
*HFA1* Stop −378 5′-3′	CTCGCCGGATACATTCTCACTTTTACATTCCCATATTCACAATTA
*HFA1* Stop −378 3′-5′	TAATTGTGAATATGGGAATGTAAAAGTGAGAATGTATCCGGCGAG
*HFA1* Stop −375 5′-3′	GGATACATTCTCACTTTTACATTACCATATAGACAATTACTTGCATTCGAATAGG
*HFA1* Stop −375 3′-5′	CCTATTCGAATGCAAGTAATTGTCTATATGGTAATGTAAAAGTGAGAATGTATCC
*HFA1* Stop −372 5′-3′	CATTACCATATTCACATAAACTTGCATTCGAATAG
*HFA1* Stop −372 3′-5′	TAAAAGTGAGAATGTATCCGG
*HFA1* Stop −363 5′-3′	TTCACAATTACTTGCTAACGAATAGGACAGTAC
*HFA1* Stop −363 3′-5′	TATGGTAATGTAAAAGTGTGAGAATGTACCG
*HFA1* Stop −360 5′-3′	ATATTCACAATTACTTGCATTTAGATAGGACAGTAC
*HFA1* Stop −360 3′-5′	GTACTGTCCTATCTAAATGCAAGTAATTGTGAATAT
*HFA1* Stop −312- 5′-3″	GGATCCTTATCGATTCTAGAATATAACGGGCAGCCAGATAGTACGG
*HFA1* Stop −312 3′-5′	CCGTACTATCTGGCTGCCCGTTATATTCTAGAATCGATAAGGATCC
*HFA1* Stop −282 5′-3″	GCAGCCAGATAGTACGGTAGAAAGGACAGCGCC
*HFA1* Stop −282 3′-5′	GGCGCTGTCCTTTCTACCGTACTATCTGGCTGC
*HFA1*-prom-5′ *Sma1*/*Xma1*	TTATCCCGGGCGTTCTTCGATACCCTGTCG
*HFA1*-transcr_Start seq. 3′ new	CGCGAGCTTGTACTGTCCTATTCG
*HFA1*-980 5′ HindIII	CCCAAGCTTGTTGTACACAGCAGAGTG
*HFA1*+2450 3′ *PstI*	AAACTGCAGGGCAACATAAACTACATGAT
*HFA1*+2450 5′ *PstI*	AAACTGCAGCCCACTTCTTAAAATTAGTGAA
*HFA1*+350 5′seq	GGGTGACAAGATTTCTTCC
*HFA1*+950 5′ seq	CGAATACTTATATTCACCAAAAG
*HFA1*+1450 5′ seq	CGACTGGTTGGTTAGATG
*HFA1*+2000 5′ seq	GAAAAGTGATGGTGTAATTG
*HFA1*+3000 5′ seq	GATACAACTTCAGGATTTGTTC
*HFA1*+3500 5′ seq	CGTATTACATTTGCATTTATC
*HFA1*+4000 5′ seq	GAGTGCCTAGAAACAAAGAAG
*HFA1*+4500 5′ seq	CGAGAGAAGATTTGTTTTTTG
*HFA1*+5000 5′ seq	CTAACCTAACAAATTGGCG
*HFA1*+5500 5′ seq	CGTTCAAAACAGCTCAAAC
*HFA1*+6000 5′ seq	GATAGATCGACTAGGATGC
*HFA1*+200 3′ seeq	GACCGCATCCACATCC
HindIII*ADH1*prom 5′	GATCAAGCTTGATATCCTTTTGTTGTTTCC
*ADH1* prom XbaI 3′	GATCTCTAGAAGTTGATTGTATGCTTGGTATAGC
*ADH* promoter *klac ACC SpeI-F*	CTTTTTCTGCACAATATTTCAAGCTATACCAAGCATACAATCAACT ACTAGTAGGTTAAAGAAAGTTTTG
ADH promoter klac-minusMTS ACC SpeI- F	CTTTTTCTGCACAATATTTCAAGCTATACCAAGCATACAATCAACTACTAGTATGAGTGAGGAAAATCTTTC
*YCp33-EcoRI-Klac ACC R*	ACGACGGCCAGTGAATTCGAGCTCGGTACCCGGGGATCCTCTAGAGAATTCCAATCATATCTTTATT
*ADH1*promseq −90 → −68	GTCATTGTTCTCGTTCCCTTTC
*Klac pcr R check2*	GCCTTATCCACAGTGTTCAG

### Isolation of yeast genomic DNA

Genomic DNA was extracted following the standard protocol [14].

### Disruption of the *HFA1* gene

Initial attempts to generate the *hfa1* deletion in the W1536 8B background consistently resulted in *rho^−^* derivatives. Subsequently, a strain carrying a wild type copy of *HFA1* on a plasmid was used to prevent generation of a rho^−^ mutant in the absence of *HFA1.* The W1536 8B Δ*hfa1* pTSV30 *HFA1* strain was generated from W1536 8B carrying pTSV30 *HFA1* by transformation with a PCR product containing the *hfa1::kanMX4* cassette amplified from the genomic DNA of BY4741 Δ*hfa1.*The transformation was carried out using a high efficiency transformation protocol [15] followed by selection for geneticin resistance. The W1536 8B Δ*hfa1* strain was obtained by allowing loss of the pTSV30 *HFA1* plasmid on glucose. Colonies lacking the plasmid were identified by white colony color. In absence of the plasmid, the strain is geneticin resistant and can be complemented by plasmid-borne wild type *HFA1*. Similar to the previous report [6] the Δ*hfa1* strain shows differences in growth behavior depending on which non-fermentable carbon source is used. The W1536 8B Δ*hfa1* strain we generated is able to weakly grow on glycerol but unable to grow on lactate at elevated temperature (33°C) (data not shown).

### Identification of the translation start site, “Stop codon scanning”

The idea of “stop codon scanning” is to pinpoint the exact translation start site by introducing in-frame stop codons in the promoter region and by monitoring the phenotype for the respiratory deficiency. An in-frame stop codon will lead to a growth deficient mutant phenotype on lactate medium if introduced downstream of the translation start site and should have no effect on the phenotype if located upstream of the translation start site. The stop codons were introduced from the region close to the location of the respiratory deficient stop codon mutation site towards upstream. We took care to design the changes in a manner so that in most cases a single base pair mutation led to an in-frame stop codon. The purpose was to make the smallest change necessary to minimize the possibility of disrupting a regulatory RNA structure or protein binding sites. Site directed mutagenesis was employed. The deletion strain W1536 8B Δ*hfa1* was transformed using the One Step Transformation protocol [16] with STOP-codon-mutagenized or control plasmids and incubated on selective media at 30°C. Transformants carrying the plasmids were then streaked for single colonies on SCL plates as well as SCD control plates and incubated at 33°C for four to seven days to test whether the plasmids were able to rescue the respiratory deficient phenotype of the deletion strain. The W1536 8B Δ*hfa1* YC*p*33 *HFA1* and W1536 8B strains were used as positive control on the non-fermentable carbon source, while the respiratory deficient W1536 8B Δ*etr1* strain, lacking the mitochondrial thioester reductase of mtFAS [17], served as a negative control. For yeast serial dilution/spotting assays, the transformants carrying the relevant constructs were first grown over night on 2 ml SCD-Ura liquid medium. The next day, the cultures were re-inoculated in 5 ml fresh SCD-Ura medium and grown for five hours to reach and OD_600_ of approximately 0.5. The OD_600_ of the individual cultures was measured, and cell concentrations were all normalized to OD_600_ = 0.5. These 1x cell suspensions were diluted to 1/10, 1/100 and 1/1000 in sterile water and spotted on SCD and SCLactate plates. The plates were then incubated for two (SCD) or three days (SCL), when the growth was then documented.

### Identification of the transcription start site with 5′RACE study

The 5′RACE experiment was done according to the protocol of the manufacturer (5′/3′ RACE Kit, 2^nd^ Generation, Roche Applied Science, Mannheim, Germany). Total RNA isolated from W1536 8B was used as template for cDNA synthesis primed by the *HFA1* specific primer SP1 (Table 3). The ss cDNA was treated with a terminal transferase that introduced a polyA tail at the 3′-end of the ss cDNA. A kit-specific polyT PCR anchor primer and a *HFA1* specific SP2 primer were used to amplify the ss cDNA by using the DyNAzymeII polymerase (Fermentas, Helsinki, Finland).

### Analysis of the *K. lactis* ACC nucleotide sequence

The *in silico* translation analysis of the 5′-upstream of the *K. lactis ACC* was performed to test for the possible presence of a mitochondrial targeting signal. We have used the MitoProtII [18] and Target P mitochondrial localization prediction programs [19] for the analysis.

### Rescue of the respiratory deficiency of the Δ*hfa*1 strain with a *K.lactis ACC* complementation construct

The translated amino acid sequence of the *K. lactis ACC* open reading frame (KLLA0F06072g) including the 5′ sequence preceding the designated start codon was obtained with the Expasy translate tool (http://web.expasy.org). The 5′ region upstream of the starting AUG of the sequence was translated up to the first in-frame STOP codon. This putative 83 amino acid sequence was analyzed with the MitoProtII program [18] and calculated for the mitochondrial targeting signal (MTS) probability. No colony developed from *Escherichia coli* cells transformed with plasmids ligated to *K. lactis ACC1*, indicating that this ORF was toxic to the bacteria. To overcome this problem, we took a yeast gap repair approach to produce the constructs directly in the yeast cells [20]. *S. cerevisiae* is able to repair gapped plasmids to circles if it is provided with a “patch” sequence that carries homology to the free plasmid arms on each end. This feature can be used to insert DNA molecules, carrying plasmid homology introduced to the ends by PCR, into linearized plasmids by co-transformation. Repaired plasmids can be identified by the plasmid nutritional marker. To control against transformation due to incompletely gapped plasmids, parallel mock transformations were carried out using only the gapped plasmid, but no insert with homologous ends. These control transformations should yield in no or much lower numbers of transformant colonies on the selective plates. The construct containing the predicted non-AUG initiated MTS together with the *K.lactis ACC* ORF was amplified with the primers ADH promoter klacACC SpeI-F and YCp33-EcoRI-klacACC-R. The *K.lactis ACC* ORF was amplified with the primer ADH promoter klac-minusMTS ACC SpeI-F and YCp33-EcoRI-klactisACC-R using genomic DNA of *K.lactis* as template (provided by Dr. Ana Rodriguez-Torres from Universidade da Coruña, Spain) and introducing 40-45 bp of homology to the *ADH1* promoter at the 5′ end and the YCplac33 multiple cloning site. Plasmid YCp33 ADH promoter was digested with XbaI and EcoRI. The PCR amplified fragments and the digested vector were transformed into the W1536 8B Δ*hfa1* strain using the high frequency transformation method [15]. Candidate colonies carrying the repaired plasmids were picked up from SCD-Ura plates and then streaked for single colonies on SCL plates and incubated at 33°C for four to seven days to test whether the plasmids were able to rescue the respiratory deficient phenotype of the deletion strain. A growth control on SCD-Ura media was performed as well. As positive control on the non-fermentable carbon source the strains W1536 8B Δ*hfa1* YC*p*33 *HFA1* and W1536 8B were used. The strain W1536 8B Δ*etr1* served as a negative, respiratory deficient control with an mtFAS defect. After the positive growth on SCL plate at 33°C was confirmed, the genomic DNA and the plasmid DNA of the candidate colonies were isolated with the yeast DNA miniprep method. PCR was performed with the *ADH1*promseq −90→ −68 primer and Klac pcr R check primer to confirm that the *K. lactis* ORF was present in these cells and fused to the *ADH1* promoter. After the correct size product was produced, the PCR product encompassing the 3′ end of the *ADH1* promoter, the fusion junction and the 5′ end of *K.lactis ACC1* was sequenced and confirmed.

### *In silico* analysis of fungal ACC evolution

**Figure 1:**
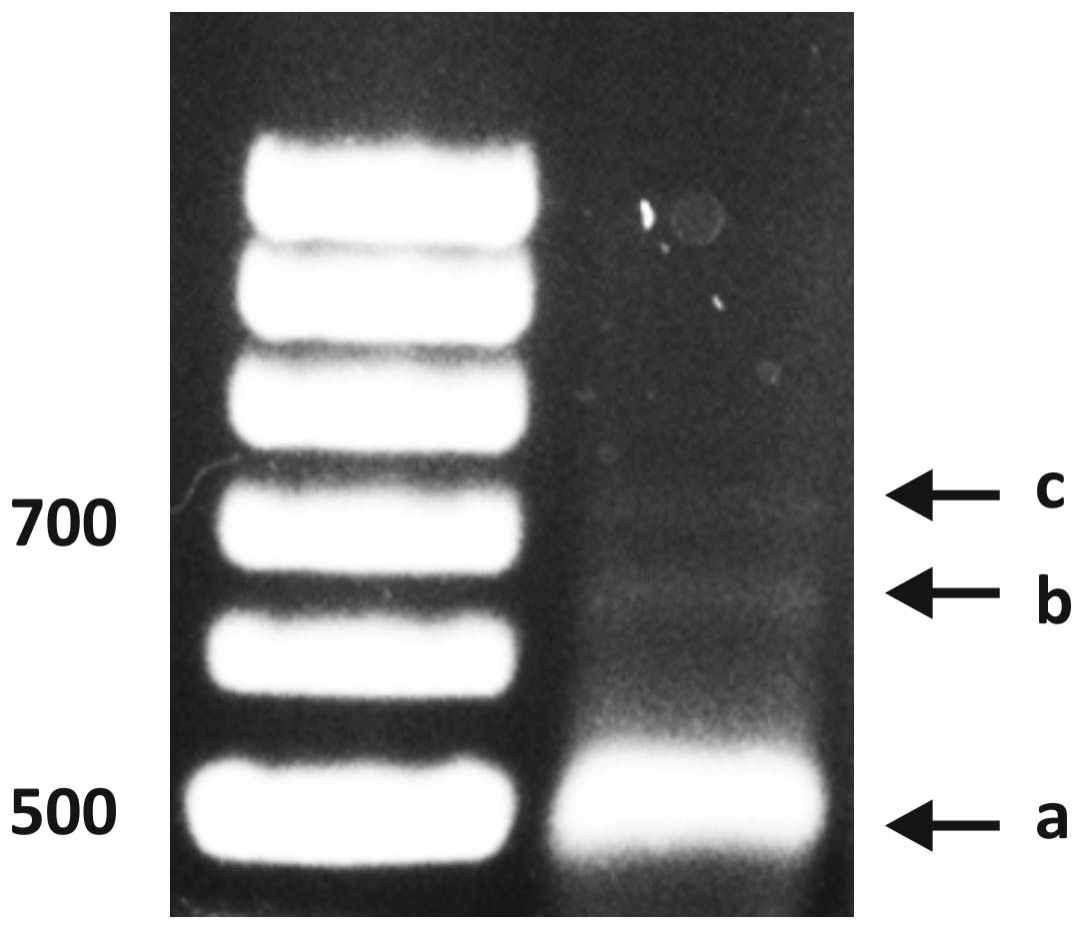
Identification of the *HFA1* transcription initiation site by 5′RACE: The *HFA1* specific primer SP1 (Table 4) was used for the reverse transcription reaction to synthesize a first strand cDNA. a) Major 5′ RACE product (500 bp), b) Minor 5′RACE product (650 bp) and c) third band produced only occasionally.

Amino acid sequences of the fungal acetyl-CoA carboxylases are obtained from the KEGG database (http://www.kegg.jp/). The phylogenetic tree shown in Fig. 1 was rooted by using aligned amino acid sequences by ClustalW2-Phylogeny [21] or ETH Phylogenetic Tree (http://www.cbrg.ethz.ch/services/PhylogeneticTree). The probability of the MTS of each amino acid sequences was analyzed with MitoProtII [18]. The 5′end of the nucleotide sequence was obtained from KEGG (http://www.kegg.jp/), Génolevures (http://www.genolevures.org/index.html), and NCBI (http://www.ncbi.nlm.nih.gov/).

Each obtained nucleotide sequence was translated with the ExPASy translate tool (http://web.expasy.org/translate/) and the matching frame was chosen for the further analysis for the MTS. After the upstream amino acid sequence from each fungal species was obtained, the MTS was analyzed up to the first in-frame stop codon with the addition of the methionine for the convenience for the analysis with MitoProtII [18].

## Results

### Transcription initiation sites of *HFA1*

We performed 5′RACE studies of *S. cerevisiae* mRNA to unequivocally determine the *HFA1* transcription initiation site(s). Two amplified products were consistently detected by agarose gel electrophoresis; a prominent band of just below 500 bp and a faint band of about 650 bp (Fig. 1). Occasionally, we also observed a third band somewhat larger than 700 bp. The two main bands were isolated, re-amplified by PCR, subcloned into the plasmid YCp*lac*33 and then sequenced. The 5′ ends of three clones out of four from the plasmid containing the 650 bp terminated at nucleotide -587 bp upstream of the designated start codon. Another clone was found to harbor an insert with the 5′ end at −618 bp upstream. The majority of the 5′ ends sequenced from these plasmids harboring the more prominent PCR product contained the nucleotide sequence between bases −441 to −424 relative to the annotated start codon. All the products obtained from the major 5′ RACE PCR band were found to be further downstream of the transcription initiation site identified by Hoja *et al.* [6]. Changes in the mRNA sequence by hypothetical RNA editing, resulting in elimination of the stop codons or the generation of an in-frame start codon, were not observed. There was also no evidence for alternative splicing.

### *HFA1* translation initiates from a non-AUG start codon (Stop codon scanning assay)

**Figure 2:**
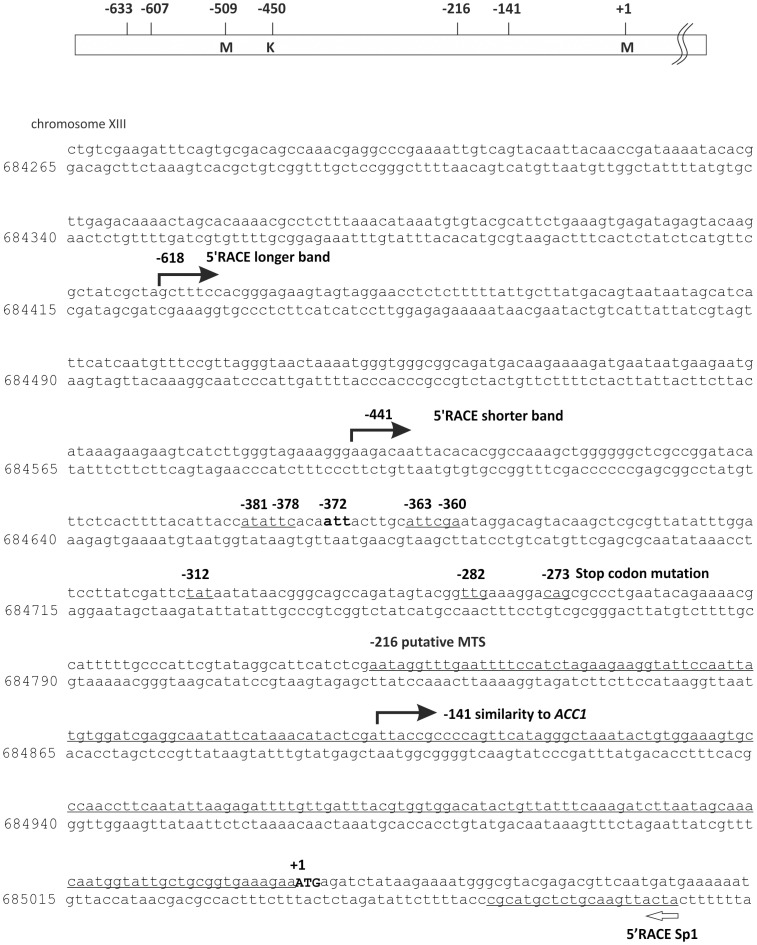
Schematic depiction of location of the introduced stop codons for stop –codon scanning assay and RNAse protection assay results. All nucleotide numbers are given respective to the annotated start codon at +1. Position −450 is the predicted transcribed but not translated region of *HFA1*. The underlined region up to position −216 region shows the putative minimum mitochondrial import sequence and upstream position −141 shows the end of the sequence similarity to *ACC1*. The ORFof *HFA1* annotated in the *Saccharomyces* Genome Database starts from +1. The stop codon found to lead to a respiratory deficient phenotype in the screen performed by Kursu *et al*. 2013 is located at −273 and 8 more stop codons at −282, −312, −360, −363, −372 −375 −378 and −381 were introduced upstream in the promoter region of *HFA1*.

**Figure 3:**
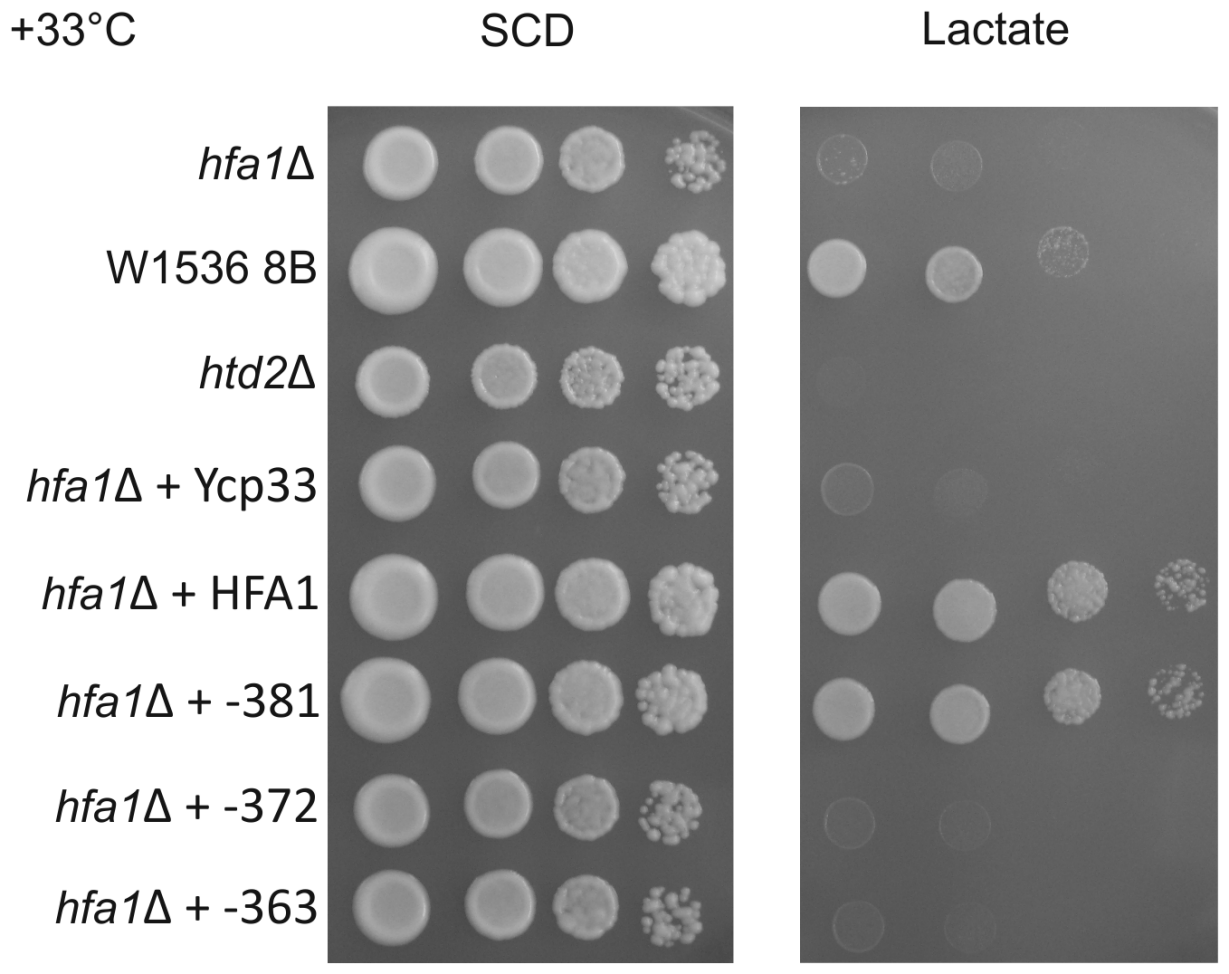
The W1536 8B Δ*hfa1* strain carrying plasmids with inserted stop codon mutation at position downstream of −372 resulted in unchanged lactate deficiency. Only stop codon mutations relevant to define the putative translation initiation site and controls are shown. The yeast cells were grown on media containing Glucose (SCD) or lactate (Lactate) as the sole carbon source at 33°C. Strains used for this study are W1536 8B, W1536 8B Δ*hfa1* or W1536 8B Δ*htd2* (respiratory deficient control) and the plasmids carried by the strains are indicated at the left side of the panels. YCp33: empty plasmid; HFA1: YCp33 *HFA1*; −381: YCp33 *HFA1* −381; −372: YCp33 *HFA1* −372; −363: YCp33*HFA1* −363. Only stop codon mutations relevant to define the putative translation initiation site and controls are shown. The results for other mutants shown in Fig. 2 can be found as supplementary data.

A mutation located upstream of the annotated start codon of *HFA1* was obtained during our study on the mtFAS in yeast seeking for respiratory deficient synthetic *petite* mutants [3]. This mutation is located upstream at −273 in frame with the designated AUG (Fig. 2). This finding further supported the hypothesis that the translation of *HFA1* mRNA does not start at the designated codon but further upstream. W1536 8B Δ*hfa1* proved to be respiratory deficient when grown on lactate as the only carbon source at a growth temperature of 33°C. None of the transformants carrying plasmids with stop codon mutations downstream of the −372 site were able to grow like the wild type strain or the Δ*hfa1* strains carrying a wild type copy of *HFA1* on the YCp33 *HFA1* plasmid. However, stop codon mutations inserted upstream of the −375 position did not impede the rescue of the respiratory deficiency of the W1536 8B Δ*hfa1* strain. The only triplet in between the mutated codons that matches the sequence of previously reported non-AUG initiation codons is ATT at position −372. The sequence context is a good match for a Kozak consensus sequence [22] (Fig. 3). The mutation of the codon in at location −372 resulted in unchanged lactate deficiency of Δ*hfa1* strains transformed with the mutagenized rescue plasmid (Fig. 3). This is not the case when the preceding likely non-AUG translation initiation codon is (−381, ATA encoding Ile) is mutated to a stop codon (Fig. 3). We therefore suggest that the native translation initiation codon of *HFA1* is ATT at −372, encoding isoleucine.

### The region 5′ to the designated starting codon of *K. lactis* ACC is required for functional complementation of the *hfa*1 mutant

**Figure 4:**
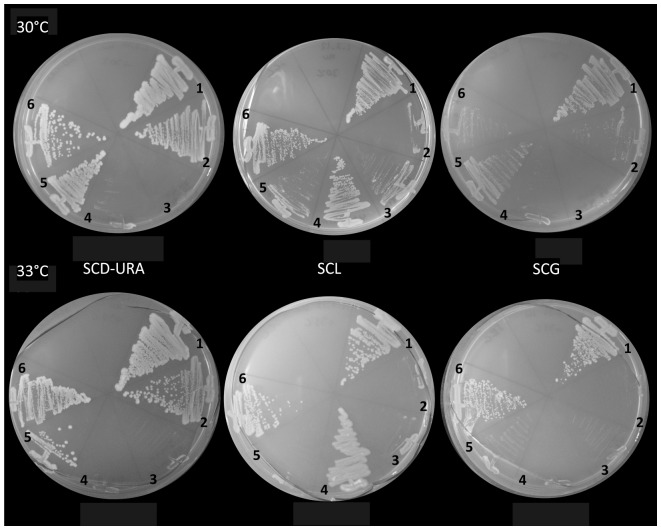
*K.lactis ACC* complemented the respiratory deficiency when appended with the putative 5′ non-AUG initiated MTS, but not without this sequence. Strains were grown on media containing glucose (SCD-URA), glycerol (SCG, non-fermentable) or lactate (SCL, non-fermentable) as the sole carbon source at 30°C and 33°C. 1) W1536 8B Δ*hfa1* +YCp33 *HFA1.* 2) W1536 8B Δ*hfa1*+YCp33 3) W1536 8B Δ*hfa1*. 4) W1536 8B (wild type). 5) W1536 8B Δ*hfa1*+*K.lactis ACC* without 5′- encoded putative cryptic MTS sequence. 6) W1536 8B Δ*hfa1*+*K.lactis ACC* with 5′ -encoded putative cryptic 5′ MTS.

Turunen *et al*. have suggested that *HFA1* and *ACC1* originated following a whole genome duplication event from a single *ACC* gene encoding a dually localized ACC [13]. Our database research confirms that *K.lactis* is predicted to have only one *ACC* (KLLA0F06072g). We analyzed the translation of the 5′-upstream region of the *K. lactis ACC* with two different subcellular localization prediction programs, MitoProtII and Target P [18], [19]. MitoProtII predicted that the region upstream to the canonical translation initiation codon encodes an in-frame protein sequence with a probability of 0.9999 to be a mitochondrial import signal, while Target P produces a mTP (mitochondrial targeting peptide) score of 0.586 for mitochondrial targeting potential. In order to add experimental evidence to the hypothesis that the dually localized, one-gene encoded variant of *ACC* is the original state of *ACC* in yeasts, we cloned and expressed the *K. lactis ACC* with and without the 5′ upstream region predicted to encode a MTS, in the *S. cerevisiae* W1536 8B Δ*hfa1* strain. Because *K. lactis* ACC was apparently toxic to *E. coli* even when expressed in low plasmid copy number, we resorted to a gap repair cloning approach in yeast (see material and methods). Functional mitochondrial localization of the *K. lacti*s ACC carrying the extra 5′ sequence was successfully demonstrated by the complementation of the respiratory deficiency of the mutant strain (12 out of 18 Ura+ candidates were respiratory competent) at 33°C. All 18 isolates from the W1536 8B Δ*hfa1* strain transformed with the *K. lactis ACC* without MTS were tested and failed to grown on the lactate plate at 33°C, suggesting that the hypothetical MTS is required for the complementation (Fig. 4).

### MTS of the ACCs are highly conserved in fungi

**Figure 5:**
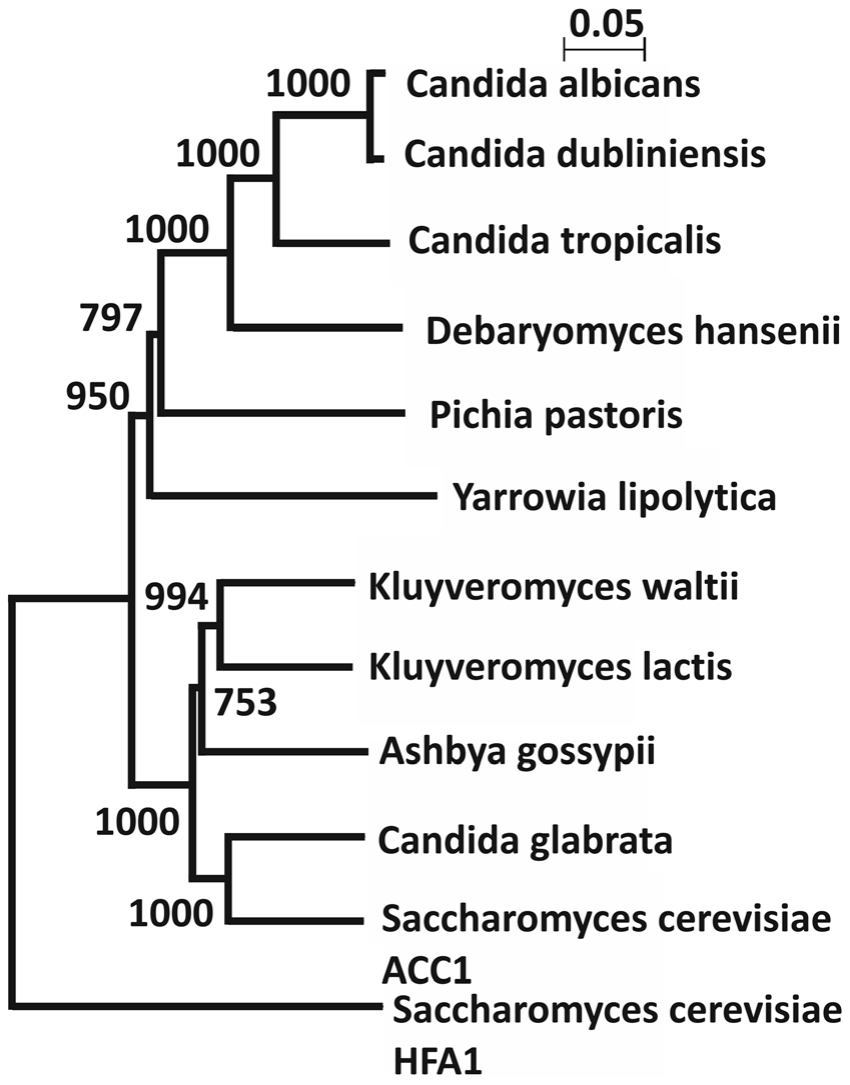
Acetyl-CoA carboxylases from yeast split into two subgroups with the exception of the *S. cerevisiae HFA1*. The phylogenetic tree of representative members of the acetyl-CoA carboxylases from fungi species was constructed using Clustal W2 Phylogeny and NJPlot as described in Material and methods. Numbers at branch points are bootstrap values and were calculated with Clustal X2.

Querying databases on the amino acid sequences of fungal ACCs, we found that the MTS is highly conserved among fungal species especially in the group of *Saccharomycotina* (Fig. 5). The alignment shows the homologous sequence of ACCs through all the fungal species, except for the N-terminal part. Our results are presented in a distance tree, displaying in the division of the fungal ACCs in two large groups (Fig. 5). One group (group 1) includes *Candida albicans, Candida dubliniensis, Candida tropicalis*, and *Debaryomyces hansenii,* and is restricted to organisms which translate CTG as serine instead of leucine. Group 2 contains the other *Saccharomyces* species, *Ashbya gossypii, Kluyveromyces waltii, K. lactis,* and two post-whole genomic duplication species, *Candida glabrata* and *S. cerevisiae*. This grouping matches with the fungal phylogeny data reported by Fitzpatrick *et al.* [23] [24]. *S. cerevisiae* Hfa1p is distantly located from the other ACCs from fungi, indicating that this protein has undergone a faster evolution compared to the other species.

The *K. waltii* ACC Kwal_6157 (KEGG entry) does not contain a MTS within the annotated ORF sequence. However, when the nucleotide sequence upstream of the ORF was investigated by a translation program, an in-frame amino acid sequence with a high probability of being a MTS (0.9923) (Table 4) was revealed.

**Table 4:** Acetyl-CoA carboxylases in yeasts and MTS probabilities.

Organism	Accession number	Group number	MTS probability(MitoProtII)/cleavage site/AA sequence length^a^	Upstream MTS probability (MitoProtII)/cleavage site/AA sequence length^b^
*C. albicans*	EEQ43196.1	1	0.8764/NP/2271	NC
*C. dubliniensis*	XP_002421671.1	1	0.0403/NP/2228	NC
*C. tropicalis*	XP_002546225.1	1	0.9935/40/2275	NC
*D. hansenii*	XP_457211.1	1	0.9693/NP/2297	NC
*P. pastoris*	FN392319	1	0.0793/NP/2215	0.9050/31/2245
*Y. lipolytica*	XP_501721.1	1	0.981621/2266	NC
*S. cerevisiae HFA1*	BK006946	2	0.4993/NP/2123	0.7297/80/2248^c^
*K. waltii*	Kwal_6157 (KEGG entry)	2	0.0195/NP/2230	0.9923/19/2281
*K. lactis*	XP_455355	2	0.0201/NP/2231	0.9999/90/2317
*A. gossypii*	NP_982612	2	0.0321/NP/2231	0.9902/52/2298
*C. glabrata*	CR380958	2	0.0090/NP/2233	0.5589/NP/2292
*S. cerevisiae ACC1*	ACC1(Locus Tag)	2	0.0279/NP/2233	0.0452/NP/2252

a : The whole amino acid sequence of each protein was analyzed with MitoProt II. ^b^: The whole sequence with the additional 5′ upstream region of the ORF of each protein was analyzed up to the first in-frame stop codon. ^c^: The whole amino acid sequence from the 5′ end upstream start codon identified in this study was analyzed. NP: not predicted. NC: not calculated

The analysis of the MTS of these fungal ACCs by MitoProtII revealed that 6 species of group 1 show high probability of MTS (<0.70) with the exception of *C. dubliniensis* and *Pichia pastoris*. Group 2 did not reveal a high probability of the MTS with their annotated ORF (Table 4). However, the translated 5′ upstream region of these ORFs showed a high probability prediction of an in-frame MTS (<0.98). *Yarrowia lipolytica*, a fungal genus in the *Saccharomycotina*, has branched before the division of the *Candida* CTG species, is also annotated to have one ACC (YALI0C11407p). The predicted protein is found to be maintaining a MTS at the N-terminus of the ORF, while *P. pastoris*, which diverged before the formation of the CTG clade [25], has only one ACC (FN392319) and does not have an MTS at the N-terminal region (MitoProtII: 0.0793). However, also this gene displays a nucleotide sequence encoding an in-frame putative MTS in the region 5′ to the annotated start codon (MitoProtII: 0.9050).

### Cryptic non-AUG inititated MTSs of mtFAS-related proteins in yeast

A cursory survey of proteins related to mtFAS revealed at least three more likely candidates for cryptic mitochondrial import sequences (see Table 5). The *BPL1* gene encodes a biotin protein ligase/holocarboxylase required to activate both Hfa1p and Acc1p and annotated to be localized in the cytosol. Analysis of the amino acid sequence 5′ to the annotated start codon of *BPL1* revealed a high probability of functioning as an MTS. The *FAA2* gene was also found to harbor an upstream sequence encoding a putative in-frame MTS, consistent with reports that a point mutation in this region which generates a new in-frame start codon can act as a suppressor of mtFAS mutations [26]–[28]. Poignantly, the wild type Faa2 protein has also recently been detected in low abundance in mitochondria [29]. Lastly, the sequence of the *SAM2* gene, coding for isoform 2 of S-adenosylmethionine (SAM) synthase and induced during the diauxic shift encodes a putative 5′ MTS.

**Table 5:** Mitochondrial targeting prediction of selected proteins in *S. cerevisiae.*

Gene name	MTS probability annotated ORF(MitoProt II)/cleavage site/sequence length	Upstream MTS probability/cleavage site/sequence length	Upstream MTS prediction/Target P			
			mTP	SP	Loc	RC
BPL1	0.0101/not (690)	0.9329/51 (760)	0.190	0.447	S	4
FAA2	0.2275/not (744)	0.8722/21 (764)	0.283	0.074	-	4
SAM2	0.0373/not (384)	0.6337/not (431)	0.363	0.516	S	5
HFA1	0.4993/not (2123)	0.7297/80 (2248) this study	0.305	0.129	-	4
ACP1	0.9996/37 (122)	NC	0.960	0.009	M	1

The whole amino acid sequence and the whole amino acid sequence with the 5′ upstream region of the ORF of each protein was analyzed up to the first in-frame stop codon with MitoProt II. In case of the Hfa1p, the identified upstream start codon with the addition of the methionine for the convenience of the calculation with MitoProt II was used. The whole amino acid sequence with the 5′ upstream region of the ORF of each protein was analyzed up to the first in-frame stop codon with Target P. In case of Acp1p, the whole amino acid sequence was used for the calculation with Target P. **mTP, SP**: Final NN scores on which the final prediction is based. Note that the scores are not really probabilities, and they do not necessarily add to one. However, the location with the highest score is the most likely according to TargetP, and the relationship between the scores (the reliability class, see below) may be an indication of how certain the prediction is. **Loc: S**: Secretory pathway, i.e. the sequence contains SP, a signal peptide **M**: Mitochondrion, i.e. the sequence contains mTP, a mitochondrial targeting peptide; -: Any other location; **RC**: Reliability class, from 1 to 5, where 1 indicates the strongest prediction. RC is a measure of the size of the difference ('diff') between the highest (winning) and the second highest output scores. There are 5 reliability classes, defined as follows: 1: diff>0.800, 2: 0.800>diff>0.600, 3: 0.600> diff>0.400, 4: 0.400> diff>0.200, 5: 0.200> diff HFA1 and ACP1 are listed as controls for the prediction calculation of MitoProt II and target P.

The proteins required for or interacting with the mtFAS pathway are listed at the KEGG as the orthologs of *S. cerevisiae BPL1, FAA2* and *SAM2* showed highly conserved putative 5′ MTS (Table 6). What was found in common in the protein sequences in between *S. cerevisiae*, *K. lactis* and *C. dubliniensis* is the significantly higher probability of a 5′ upstream encoded MTS in comparison to the MTS calculated from the annotated start codon. Other fungi species shown on Table 6 were also found to carry 5′ upstream sequences predicted to be MTSs with high probability, but not all species showed the exact match in all these three proteins, indicating diversification during the molecular evolution. For example, *C. glabrata* carries two genes encoding hypothetical proteins with similarity to SAM synthases, one with low probability of harboring an MTS, the other one with higher probability if the 5′ upstream region is included in the analysis. The region 5′ to the annotated AUG and translated in frame with the SAM ORF revealed a putative MTS with a predicted cleavage site. *C. glabrata* is one of the fungal species which has undergone the whole genomic duplication. This could be another example of the molecular evolution of one protein gave rise to divergence of localization of paralogs after the genomic duplication. Other examples supporting this hypothesis are found in the pre-genomic duplication species such as *K. lactis*, *A. gossypii* and *C. dubliniensis*. These species carry a single gene encoded variants of these proteins with putative MTSs either or both in the 5′ sequence and annotated sequence.

**Table 6:** Mitochondrial targeting prediction of fungal homologues of proteins in Table 5.

Gene name	Organism	Entry	Definition	MTS probability/cleavage site/AA sequence length^a^	Upstream MTS probability/cleavage site/AA sequence length^b^
*BPL1*	*S. cerevisiae*	YDL141W	Biotin:apoprotein ligase	0.0101/NP/690	0.9329/51/760
	*K. lactis*	KLLA0F05049g	hypothetical protein	0.0477/NP/693	0.9870/30/732
	*A. gossypii*	AGOS_ADR085W	hypothetical protein	0.0314/NP/679	0.6205/NP/720
	*C. dubliniensis*	CD36_35420	biotin: apoprotein ligase	0.0108/NP/665	0.6348/NP/689
	*Z. rouxii*	ZYRO0F05940g	hypothetical protein	0.0219/NP/690	0.8872/30/722
	*L.thermotolerans*	KLTH0H02024g	biotin−protein ligase	0.042/NP/660	0.4203/53/7110
	*K. africana*	KAFR_0B01050	hypothetical protein	0.0219/NP/690	0.8872/30/722
*FAA2*	*S. cerevisiae*	YER015W	medium-chain fatty acid-CoA ligase FAA2	0.1550/NP/744	0.8807/21/765
	*K. lactis*	KLLA0B11572g	long-chain fatty acyl-CoA synthetase	0.1983/NP/745	0.2603/NP/749
	*C. glabrata*	CAGL0H09460g	hypothetical protein	0.3174/NP/741	0.4203/NP/752
	*T. delbureckii*	TDEL_0H02790	hypothetical protein	0.2323/NP/741	0.8615/NP/750
*SAM2*	*S. cerevisiae*	YDR502c	S-adenosylmethionine synthetase	0.0373/NP/384	0.6377/NP/430
	*K. lactis*	KLLA0C01782g	hypothetical protein	0.0159/NP/384	0.1565/NP/392
	*A. gossypii*	AGOS_AFR692C	AFR692Cp	0.3994/14/382	0.1639/NP/387
	*Z. rouxii*	ZYRO0F17248g	hypothetical protein	0.0769/NP/382	0.3633/NP/412
	*P. pastoris*	PAS_chr3_0876	S-adenosylmethionine synthetase	0.0139/NP/384	0.5093/NP/423*^1^
	*C. glabrata*	CAGL0J08415g	hypothetical protein	0.0634/15/383	0.2737/29/397*^2^
	*C. glabrata*	CAGL0B01122g	hypothetical protein	0.0727/NP/382	0.0308/NP/394
	*C. dubliniensis*	CD36_10740	S-adenosylmethionine synthetase, putative	0.0280/NP/385	0.9093/58/447

a : The whole amino acid sequence of each protein was analyzed with MitoProt II. ^b^: The 5′ upstream region of the ORF of each protein was analyzed up to the first in-frame stop codon. *^1^The *P. pastoris SAM* homolog is preceded by a 39 aa sequence with high probability of being an MTS and starting with a *bona fide* ATG that apparently has been omitted in the annotation, probably because this sequence does not show homology with the *S. cerevisiae SAM1* or *SAM2* genes. *^2^ Translation from the first likely initiation codon (ATA, Ile) results in a probability of 0.4938.

## Discussion

*HFA1* clearly does not produce a functional protein from the annotated ATG in the yeast database, and the *in vivo* translation initiation site is not known [6]. In addition, the existing reports on the transcription initiation site on the *HFA1* mRNA showed disagreement in the 5′ extent of the transcript, placing it 633 to 607 base pairs [6], or 534 base pairs for the poly(a)RNA array and 430 base pairs for the total RNA array 5′ [30] relative to the annotated translation initiation AUG codon. The transcript reported by Hoja *et al.* would contain an AUG start codon in frame with the *HFA1* coding region, which is soon followed by two stop codons. The variants identified by Fitzpatrick *et al.* would not contain any upstream in-frame AUG at all [24]. In this work, we have demonstrated that at least two *HFA1* transcripts exist, with the major mRNA species being the shorter form initiated between nucleotides −442 → −435. The sequence analysis of the transcription products confirmed that there is no RNA editing. The existence of this major shorter transcript also makes a frame-shifting explanation unnecessary. In essence, both existing reports on the transcription start sites of *HFA1* are correct [6], [30], but our results indicated that the most proximal to the annotated AUG is likely to be the physiologically most relevant one.

In this study, we have pinpointed the exact location of the translation initiation site using a “stop codon scanning” approach, which reveals the likely non-AUG start codon usage of this gene. This expression principle resembles the previously reported situation of tRNA synthetases, where the mitochondrial versions of dually localized proteins were described to be translated from non-AUG codons [10], [11]. In yeast, a redundancy of non-AUG initiation codons were reported for *ALA1* [31] and *GRS1* [32]. The non-AUG codons for these genes act as alternative translation initiation sites with downstream in-frame AUG initiation codons, which can be observed in our *K.lactis* model described in the paragraph below. Our stop codon scanning results also argue against a major role of the upstream ATGs in translation.

We chose the *K. lactis ACC* gene as a model to investigate the evolutionary history of *S. cerevisiae* ACC isoforms. In this study, we showed that the only ACC encoded by the *K. lactis* genome was capable of complementing the respiratory deficiency of an Δ*hfa1* strain only if the construct included the 5′ upstream region of the *K. lactis* gene, encoding a putative MTS. This result supports the hypothesis that the single *K. lactis ACC* gene encodes both the cytosolic and mitochondrial isoforms of the enzyme.

Using a combination of bioinformatics methodologies and a genetic approach, our results illustrate an important aspect of the evolution of one mitochondrial protein instigated by a whole genome duplication (WGD). A recent study on the comparative genomics of yeast revealed how the genomic evolution has been affected by the WGD [33]. Genomic duplications are considered to be an important source of evolutional novelty, providing extra genetic material that is free to diverge without compromising the organism, as a copy retaining the original function exists [34]. Not only fungi but also other higher eukaryotic genomes show a high degree of redundancy [35], [36] [33]. Wolfe and Shields proposed a model of massive gene deletion in the wake of the gene duplication in yeast [37]. By systematic study of the genomic data from *K. lactis* and *S. cerevisiae* targeting the duplicated segments of the *S. cerevisiae* genome, they have shown that most of the duplicated genes were lost. The remaining small fractions of the genes were rearranged by many reciprocal translocations between chromosomes. A preceding study has shown that duplicated genes are almost as likely to acquire a new and essential function as to be lost through acquisition of mutations that compromise protein function [35], [38]. This was also confirmed by Kellis *et al.* [12] in their study describing how the genome duplication of *S. cerevisiae* was followed by random local deletion of genes during the course of evolution. The authors propose that the paralogs derived from the genome duplication have specialized in their cellular localization or temporal expression. They take *ACC1* and *HFA1*as one example pair and describe that the former gave rise to the latter one, suggesting a model that the ancestral cytoplasmic form acquired the MTS after the gene duplication event. The conclusions of Hoja *et al.* [6], who published their results on the analysis of *HFA1* almost simultaneously, do not state the same hypothesis explicitly, but echo a similar conclusion. In contrast, an *in silico* study on functional specialization events after WGD in yeast reports that the new localization patterns have evolved in the duplicated genes [13]. They show *ACC1* as one example that has lost the MTS, whereas *HFA1* retained it through evolution. Our results support the *in silico* data, and suggest that the MTS of fungal ACC was gained long before the gene duplication event that generated *S. cerevisiae*. The views of Kellis *et al.* and Hoja *et al.* on the origin of present-day baker's yeast ACCs are not supported by our data. Our experimental data is much more consistent with the *in silico* results by Turunen *et al.*, indicating that both ACC forms are derived from a dually localized ancestral gene and adapted to serve their more specialized roles in different compartments.

Our phylogenetic study on the amino acid composition of the ACCs in *Saccharomycotina* clarified that the ACCs are categorized in to two large groups, one is the CTG clade (group1) and the other one contains the WGD species and *Saccharomycotina* (group2) which have not undergone WGD. This study based on the protein sequence of the fungal ACCs matches to the fungal phylogeny based on the complete genome analysis [23]. Additional data that we obtained from the analysis of the MTS probability of the ACCs supports this grouping. All the group1 species carry ACCs with high MTS probability (<0.7) with the exception of the *C. dubliniensis* and *P. pastoris* and all the group 2 species except *S. cerevisiae* carry a putative MTS (<0.9) in the upstream of the start codon. This phylogenetic analysis and MTS conservation in fungi also suggests that the ancestral gene encoding acetyl-CoA carboxylase allowed the production of two proteins, one with a MTS which is almost always initiated from a non-AUG translation start codon, and a cytosolic variant of which translation starts from a canonical AUG. Hfa1p is distantly localized in the phylogenetic tree in this study, indicating that the evolution of this protein is faster than the other ACCs in fungi, possibly to allow specialization for the mitochondrial compartment. The favorable MTS of the ACC upstream of the start codon is also conserved in fungi species diverged before the formation of the CTG clade, supporting our hypothesis that the Hfa1p, carrying MTS is the original form and Acc1p arose after WGD by deleting the MTS.

A recent report on the *GAL1*/*GAL3* gene duplication showed that specialization of proteins encoded by gene pairs can increase the evolutionary fitness of an organism, as the dual use of a product encoded by a single gene can imply compromise in function [39]. Our limited analysis of a small subset of genes of our interest resulted in a few candidates with a possible concealed mitochondrial localization. The obvious requirement for Bpl1p in mitochondria to activate Hfa1p is probably the clearest support for a physiological role of this putatively mitochondrial isoform. Results on the mitochondrial localization of human or mouse biotin protein ligase/holocarboxylase are ambiguous [40], [41] and deserve a more thorough investigation. The mitochondrial roles of the other two candidates are less clear cut. There is support in the literature on mitochondrial localization of Faa2p [29]. The most controversial of our candidates may be *SAM2*, as mitochondria are clearly dependent on import of SAM under normal growth conditions [42]. Intriguingly, the same publication demonstrated that, with exception of SAM synthase itself, the entire SAM recycling machinery is present in mitochondria. It may not be impossible to find a condition where cytosolic SAM is of too short supply to support the mitochondrial demand of this cofactor. Poignantly, one of each of the two versions of duplicated SAM synthase genes (*MAT2A/Mat2a*) in both the mouse and human genomes also potentially encodes a putative 5′-encoded non-AUG initiated MTSs (unpublished).

## Supporting Information

S1 Figure **Stop codon scanning mutant phenotype.** The W1536 8B Δ*hfa1* strain carrying plasmids with inserted stop codon mutation at position −282, −312, −360, and −378 were plated onto the indicated media under the indicated growth temperatures to show the complementation or lack thereof of the different stop codon mutant constructs: The carbon sources of the media are Glucose (SCD, fermentable, left panel) or lactate (SCL, non-fermentable, middle and right panel) as the sole carbon source and cultured at 30°C and 33°C. The strains/transformants plated are as follows: 1. W1536 8B wild type, 2. W1536 8B Δ*hfa1*+pTSV30 *HFA1* (multicopy plasmid), 3. W1536 8B Δ*etr1* (respiratory deficient control), 4. W1536 8B Δ*hfa1,* 5. W1536 8B Δ*hfa1*+YCp33 (empty plasmid), 6. W1536 8B Δ*hfa1*+YCp33 *HFA1*, 7–10. W1536 8B Δ*hfa1* carrying the indicated stop codon plasmids (four independent transformants). a. W1536 8B wild type, b. W1536 8B Δ*hfa1,* c. W1536 8B Δ*etr1* (respiratory deficient control), d. W1536 8B Δ*hfa1*+YCp33 *HFA1,* e. W1536 8B Δ*hfa1*+YCp33 (empty plasmid), f-h. W1536 8B Δ*hfa1* colonies carrying the indicated stop codon plasmids (three independent transformants).
(TIF)

## References

[1] Schonauer MS, Kastaniotis AJ, Hiltunen JK, Dieckmann CL (2008) Intersection of RNA processing and the type II fatty acid synthesis pathway in yeast mitochondria. Mol Cell Biol 28:6646–6657. 18779316 10.1128/MCB.01162-08 PMC2573234

[2] Hiltunen JK, Autio KJ, Schonauer MS, Kursu VA, Dieckmann CL, et al (2010) Mitochondrial fatty acid synthesis and respiration. Biochim Biophys Acta 1797:1195–1202. 20226757 10.1016/j.bbabio.2010.03.006

[3] Kursu VA, Pietikainen LP, Fontanesi F, Aaltonen MJ, Suomi F, et al (2013) Defects in mitochondrial fatty acid synthesis result in failure of multiple aspects of mitochondrial biogenesis in saccharomyces cerevisiae. Mol Microbiol 90:824–840. 24102902 10.1111/mmi.12402 PMC4153884

[4] Saggerson D (2008) Malonyl-CoA, a key signaling molecule in mammalian cells. Annu Rev Nutr 28:253–272. 18598135 10.1146/annurev.nutr.28.061807.155434

[5] Hasslacher M, Ivessa AS, Paltauf F, Kohlwein SD (1993) Acetyl-CoA carboxylase from yeast is an essential enzyme and is regulated by factors that control phospholipid metabolism. J Biol Chem 268:10946–10952. 8098706

[6] Hoja U, Marthol S, Hofmann J, Stegner S, Schulz R, et al (2004) HFA1 encoding an organelle-specific acetyl-CoA carboxylase controls mitochondrial fatty acid synthesis in saccharomyces cerevisiae. J Biol Chem 279:21779–21786. 14761959 10.1074/jbc.M401071200

[7] Zitomer RS, Walthall DA, Rymond BC, Hollenberg CP (1984) Saccharomyces cerevisiae ribosomes recognize non-AUG initiation codons. Mol Cell Biol 4:1191–1197. 6390186 10.1128/mcb.4.7.1191-1197.1984 PMC368898

[8] Natsoulis G, Hilger F, Fink GR (1986) The HTS1 gene encodes both the cytoplasmic and mitochondrial histidine tRNA synthetases of S. cerevisiae. Cell 46:235–243. 3521891 10.1016/0092-8674(86)90740-3

[9] Chatton B, Walter P, Ebel JP, Lacroute F, Fasiolo F (1988) The yeast VAS1 gene encodes both mitochondrial and cytoplasmic valyl-tRNA synthetases. J Biol Chem 263:52–57. 3275649

[10] Tang HL, Yeh LS, Chen NK, Ripmaster T, Schimmel P, et al (2004) Translation of a yeast mitochondrial tRNA synthetase initiated at redundant non-AUG codons. J Biol Chem 279:49656–49663. 15358761 10.1074/jbc.M408081200

[11] Chang KJ, Wang CC (2004) Translation initiation from a naturally occurring non-AUG codon in saccharomyces cerevisiae. J Biol Chem 279:13778–13785. 14734560 10.1074/jbc.M311269200

[12] Kellis M, Birren BW, Lander ES (2004) Proof and evolutionary analysis of ancient genome duplication in the yeast saccharomyces cerevisiae. Nature 428:617–624. 15004568 10.1038/nature02424

[13] Turunen O, Seelke R, Macosko J (2009) In silico evidence for functional specialization after genome duplication in yeast. FEMS Yeast Res 9:16–31. 19133069 10.1111/j.1567-1364.2008.00451.x PMC2704937

[14] Kaiser C, Michaelis S, Mitchell A (1994) Methods in yeast genetics: A cold spring harbor laboratory course manual. New York: Cold Spring Harbor Laboratory Press.

[15] Gietz RD, Woods RA (2002) Transformation of yeast by lithium acetate/single-stranded carrier DNA/polyethylene glycol method. Methods Enzymol 350:87–96. 12073338 10.1016/s0076-6879(02)50957-5

[16] Chen DC, Yang BC, Kuo TT (1992) One-step transformation of yeast in stationary phase. Curr Genet 21:83–84. 1735128 10.1007/BF00318659

[17] Torkko JM, Koivuranta KT, Miinalainen IJ, Yagi AI, Schmitz W, et al (2001) Candida tropicalis Etr1p and saccharomyces cerevisiae Ybr026p (Mrf1'p), 2-enoyl thioester reductases essential for mitochondrial respiratory competence. Mol Cell Biol 21:6243–6253. 11509667 10.1128/MCB.21.18.6243-6253.2001 PMC87346

[18] Claros MG, Vincens P (1996) Computational method to predict mitochondrially imported proteins and their targeting sequences. Eur J Biochem 241:779–786. 8944766 10.1111/j.1432-1033.1996.00779.x

[19] Emanuelsson O, Brunak S, von Heijne G, Nielsen H (2007) Locating proteins in the cell using TargetP, SignalP and related tools. Nat Protoc 2:953–971. 17446895 10.1038/nprot.2007.131

[20] Kostrub CF, Lei EP, Enoch T (1998) Use of gap repair in fission yeast to obtain novel alleles of specific genes. Nucleic Acids Res 26:4783–4784. 9753750 10.1093/nar/26.20.4783 PMC147907

[21] Larkin MA, Blackshields G, Brown NP, Chenna R, McGettigan PA, et al (2007) Clustal W and clustal X version 2.0. Bioinformatics 23:2947–2948. 17846036 10.1093/bioinformatics/btm404

[22] Kozak M (1984) Point mutations close to the AUG initiator codon affect the efficiency of translation of rat preproinsulin in vivo. Nature 308:241–246. 6700727 10.1038/308241a0

[23] Fitzpatrick DA, Logue ME, Stajich JE, Butler G (2006) A fungal phylogeny based on 42 complete genomes derived from supertree and combined gene analysis. BMC Evol Biol 6:99. 17121679 10.1186/1471-2148-6-99 PMC1679813

[24] Fitzpatrick DA, Creevey CJ, McInerney JO (2006) Genome phylogenies indicate a meaningful alpha-proteobacterial phylogeny and support a grouping of the mitochondria with the rickettsiales. Mol Biol Evol 23:74–85. 16151187 10.1093/molbev/msj009

[25] De Schutter K, Lin YC, Tiels P, Van Hecke A, Glinka S, et al (2009) Genome sequence of the recombinant protein production host pichia pastoris. Nat Biotechnol 27:561–566. 19465926 10.1038/nbt.1544

[26] Harington A, Herbert CJ, Tung B, Getz GS, Slonimski PP (1993) Identification of a new nuclear gene (CEM1) encoding a protein homologous to a beta-keto-acyl synthase which is essential for mitochondrial respiration in saccharomyces cerevisiae. Mol Microbiol 9:545–555. 8412701 10.1111/j.1365-2958.1993.tb01715.x

[27] Kastaniotis AJ, Autio KJ, Sormunen RT, Hiltunen JK (2004) Htd2p/Yhr067p is a yeast 3-hydroxyacyl-ACP dehydratase essential for mitochondrial function and morphology. Mol Microbiol 53:1407–1421. 15387819 10.1111/j.1365-2958.2004.04191.x

[28] Autio KJ, Kastaniotis AJ, Pospiech H, Miinalainen IJ, Schonauer MS, et al (2008) An ancient genetic link between vertebrate mitochondrial fatty acid synthesis and RNA processing. FASEB J 22:569–578. 17898086 10.1096/fj.07-8986

[29] Vogtle FN, Wortelkamp S, Zahedi RP, Becker D, Leidhold C, et al (2009) Global analysis of the mitochondrial N-proteome identifies a processing peptidase critical for protein stability. Cell 139:428–439. 19837041 10.1016/j.cell.2009.07.045

[30] David L, Huber W, Granovskaia M, Toedling J, Palm CJ, et al (2006) A high-resolution map of transcription in the yeast genome. Proc Natl Acad Sci U S A 103:5320–5325. 16569694 10.1073/pnas.0601091103 PMC1414796

[31] Chang KJ, Lin G, Men LC, Wang CC (2006) Redundancy of non-AUG initiators. A clever mechanism to enhance the efficiency of translation in yeast. J Biol Chem 281:7775–7783. 16431919 10.1074/jbc.M511265200

[32] Chen SJ, Lin G, Chang KJ, Yeh LS, Wang CC (2008) Translational efficiency of a non-AUG initiation codon is significantly affected by its sequence context in yeast. J Biol Chem 283:3173–3180. 18065417 10.1074/jbc.M706968200

[33] Piskur J, Langkjaer RB (2004) Yeast genome sequencing: The power of comparative genomics. Mol Microbiol 53:381–389. 15228521 10.1111/j.1365-2958.2004.04182.x

[34] Ohno S (1970) Evolution by gene duplication. Berlin, New York: Springer-Verlag. 160 p.

[35] Nadeau JH, Taylor BA (1984) Lengths of chromosomal segments conserved since divergence of man and mouse. Proc Natl Acad Sci U S A 81:814–818. 6583681 10.1073/pnas.81.3.814 PMC344928

[36] Schughart K, Kappen C, Ruddle FH (1989) Duplication of large genomic regions during the evolution of vertebrate homeobox genes. Proc Natl Acad Sci U S A 86:7067–7071. 2571149 10.1073/pnas.86.18.7067 PMC297995

[37] Wolfe KH, Shields DC (1997) Molecular evidence for an ancient duplication of the entire yeast genome. Nature 387:708–713. 9192896 10.1038/42711

[38] Nadeau JH, Sankoff D (1997) Comparable rates of gene loss and functional divergence after genome duplications early in vertebrate evolution. Genetics 147:1259–1266. 9383068 10.1093/genetics/147.3.1259 PMC1208249

[39] Hittinger CT, Carroll SB (2007) Gene duplication and the adaptive evolution of a classic genetic switch. Nature 449:677–681. 17928853 10.1038/nature06151

[40] Aoki Y, Suzuki Y, Sakamoto O, Li X, Takahashi K, et al (1995) Molecular analysis of holocarboxylase synthetase deficiency: A missense mutation and a single base deletion are predominant in japanese patients. Biochim Biophys Acta 1272:168–174. 8541348 10.1016/0925-4439(95)00082-8

[41] Bailey LM, Wallace JC, Polyak SW (2010) Holocarboxylase synthetase: Correlation of protein localisation with biological function. Arch Biochem Biophys 496:45–52. 20153287 10.1016/j.abb.2010.01.015

[42] Cherest H, Surdin-Kerjan Y (1978) S-adenosyl methionine requiring mutants in saccharomyces cerevisiae: Evidences for the existence of two methionine adenosyl transferases. Mol Gen Genet 163:153–167. 355845 10.1007/BF00267406

[43] Johnson ES, Gupta AA (2001) An E3-like factor that promotes SUMO conjugation to the yeast septins. Cell 106:735–744. 11572779 10.1016/s0092-8674(01)00491-3

[44] Menger K (2008) Study of the expression of the yeast mitochondrial acetyl-CoA carboxylase HFA1 gene. Oulu: Menger K 72, 6 p.

