# Supplementary figures and images for: Expression and Evolution of the Non-Canonically Translated Yeast Mitochondrial Acetyl-CoA Carboxylase Hfa1p
Source: PLoS One. 2014 Dec 11;9(12):e114738. doi: 10.1371/journal.pone.0114738 (PMC4263661; doi:10.1371/journal.pone.0114738)

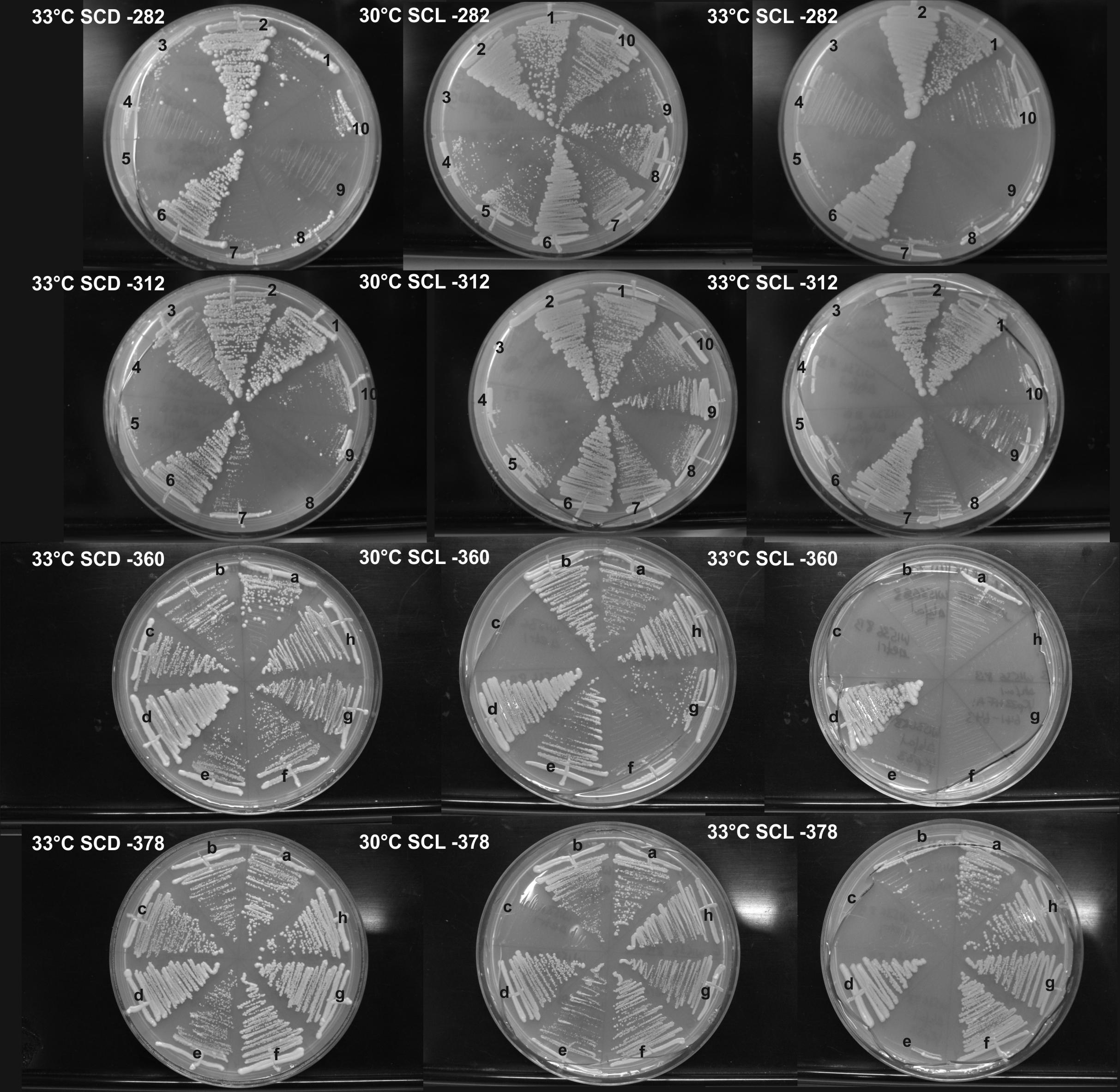

Supplement: S1 Figure — Stop codon scanning mutant phenotype. The W1536 8B Δhfa1 strain carrying plasmids with inserted stop codon mutation at position −282, −312, −360, and −378 were plated onto the indicated media under the indicated growth temperatures to show the complementation or lack thereof of the different stop codon mutant constructs: The carbon sources of the media are Glucose (SCD, fermentable, left panel) or lactate (SCL, non-fermentable, middle and right panel) as the sole carbon source and cultured at 30°C and 33°C. The strains/transformants plated are as follows: 1. W1536 8B wild type, 2. W1536 8B Δhfa1+pTSV30 HFA1 (multicopy plasmid), 3. W1536 8B Δetr1 (respiratory deficient control), 4. W1536 8B Δhfa1, 5. W1536 8B Δhfa1+YCp33 (empty plasmid), 6. W1536 8B Δhfa1+YCp33 HFA1, 7–10. W1536 8B Δhfa1 carrying the indicated stop codon plasmids (four independent transformants). a. W1536 8B wild type, b. W1536 8B Δhfa1, c. W1536 8B Δetr1 (respiratory deficient control), d. W1536 8B Δhfa1+YCp33 HFA1, e. W1536 8B Δhfa1+YCp33 (empty plasmid), f-h. W1536 8B Δhfa1 colonies carrying the indicated stop codon plasmids (three independent transformants). (TIF) [file pone.0114738.s001.tif]
